# Application of Non-*Saccharomyces* Yeast for the Production of Low-Alcohol Beer

**DOI:** 10.3390/foods13203214

**Published:** 2024-10-10

**Authors:** Krystian Klimczak, Monika Cioch-Skoneczny, Aneta Ciosek, Aleksander Poreda

**Affiliations:** Department of Fermentation Technology and Microbiology, University of Agriculture in Kraków, ul. Balicka 122, 30-149 Kraków, Poland; krystian.klimczak.sd@student.urk.edu.pl (K.K.); aneta.ciosek@urk.edu.pl (A.C.); aleksander.poreda@urk.edu.pl (A.P.)

**Keywords:** beer, non-*Saccharomyces*, low-alcohol beer

## Abstract

In recent years, demand for low-alcohol and alcohol-free beers has been rising. Of the many methods of producing such beers, many have expensive implementation requirements or drawbacks in terms of beer quality. The exploration of non-*Saccharomyces* yeast species presents a promising opportunity to overcome these challenges. These yeasts, with their diverse metabolic capabilities and unique flavor profiles, offer the potential to create innovative and flavorful low-alcohol beers. The study investigates the feasibility of using selected non-*Saccharomyces* yeasts for brewing low-alcohol beers, focusing on fermentation kinetics, physicochemical parameters, and the sensory attributes of the final product. The evaluated yeast species were *Kluyveromyces lactis* MG971263, *Metschnikowia pulcherrima* MG971247 and MG971250, *Torulaspora delbrueckii* MG971248, *Wickerhamomyces anomalus* MG971261, and *W. onychis* MG971246. Two strains of *Saccharomyces cerevisiae* were used as a control. The results of the study show that selected non-*Saccharomyces* yeast species might be used to produce low-alcohol beers. The non-*Saccharomyces* yeast allowed the researchers to obtain beers with an alcohol content in the range of 0.5–1.05%, while the control beer brewed with US-05 had an alcohol content of 3.77%. Among the evaluated strains, the strains *M. pulcherrima* MG971250 and *T. delbrueckii* MG971248 were found to be rated better in a sensory evaluation than the brewed and low-alcohol strains of *S. cerevisiae*.

## 1. Introduction

For many years, beer has been the most popular alcoholic beverage in the world [1]. However, this market is far from stationary and consolidated. The craft beer revolution has brought about many unique beer styles and new tastes. Partially as a result of that revolution, consumers are increasingly seeking new beer styles and products [2]. The ongoing popularity of beer can be seen in the data—in Europe, the total consumption of beer for the years 2016–2022 fluctuated around 365 mln hL per year [3]. However, consumer preferences constantly change, and the brewing market has to adapt to these changes [1]. Nowadays, consumers often look for lower-alcohol beverages due to health, driver safety, and lifestyle considerations [4]. The share of non-alcoholic beer in the total beer volume in Europe increased from 1.8% in 2013 to 3.8% in 2019 [5]. This trend continues today; according to Eurostat, in 2023, the production of beer with normal alcohol levels fell by 5% (−1.7 bn liters) compared to the previous year. On the contrary, the production of low-alcohol beer increased by 13.5% (+0.2 bn liters) [6].

However, these market changes have accentuated the difficulty of obtaining high-quality low-alcohol beers. The methods currently used to produce such beers can be categorized into two main types: physical and biological processes. The first group of methods focuses on the removal of alcohol from the fermented beer. The main problems with these methods are the high cost of the equipment and the substantial energy demand. Additionally, during the removal of alcohol, a significant portion of volatile compounds, which give beer its aroma, are lost [7]. An interesting alternative method of producing such beers is through the usage of special yeasts. This approach is an example of a biological method, and it focuses on using yeasts that do not ferment maltose or that do so poorly. Maltose is the primary sugar in brewers’ wort, accounting for 50–60% of the total sugars. Therefore, if maltose-negative yeasts are used, they will inherently yield a beer with lower alcohol content [8]. An additional benefit is that some of these yeasts often possess higher levels of enzymatic activities related to biotransformation or produce a distinct profile of volatile compounds [9]. One of the main problems related to traditionally produced low-alcohol beers is their inappropriate sensory characteristics, i.e., wort off-flavors and the lack of a fruity aroma [10]. The non-*Saccharomyces* yeast can be used to produce a beer without these sensory drawbacks. They might then fulfill consumer interest in new and unique products [4]. Additionally, introducing yeast species as a factor that limits alcohol levels would be easy to implement in the production of beer [11]. Another often overlooked aspect is the health benefit of selected non-*Saccharomyces* strains. Some strains with probiotic properties might be used to brew beer [12,13]. These features make non-*Saccharomyces* strains a promising way to produce alcohol-reduced beers.

However, for now, the use of non-*Saccharomyces* yeasts is mostly limited to academic research. Most of the non-*Saccharomyces* yeasts on the market are aimed at the wine industry, with only a few strains available for the brewing market [14,15,16]. The most important requirements of yeasts designed for the production of low-alcohol beers are lack of maltose fermentation, lack of phenolic off-flavors, ease of flocculation, and consumer safety [17]. The currently available research shows that many non-*Saccharomyces* yeasts fulfill those requirements. Given the growing consumer demand for low-alcohol beers and the potential of these yeasts to create innovative and flavorful products, further research in this area could bring significant benefits to both the brewing industry and consumers.

The aim of this study was the evaluation of selected yeast species in producing alcohol-reduced beer. This study focused on rating the physicochemical and sensory attributes of the obtained beers. During fermentation, the kinetics of the process were determined. In the final beers, parameters such as alcohol and extract content, apparent degree of fermentation, IBU, free amino nitrogen, color, titratable acidity, and metal ion content were determined. Additionally, the sensory characteristics of the beers were evaluated.

## 2. Materials and Methods

### 2.1. Materials

This study used 6 strains of non-*Saccharomyces* yeasts and 2 control strains of *Saccharomyces* yeasts. The non-*Saccharomyces* strains used in the study were obtained from the pure culture collection of the Department of Fermentation Technology and Microbiology of the University of Agriculture in Kraków. The yeast strains were isolated from winery environments in the Malopolska and Podkarpacie regions of Poland. They were selected for this study based on previous research [18]. The selected yeasts were maltose-negative, and as such were expected to produce low alcohol levels. The evaluated non-*Saccharomyces* yeasts were as follows:

*Kluyveromyces lactis* MG971263 (KL1), *Metschnikowia pulcherrima* MG971247 (MP1), *M. pulcherrima* MG971250 (MP2), *Torulaspora delbrueckii* MG971248 (TD1), *Wickerhamomyces anomalus* MG971261 (WA1), *W. onychis* MG971246 (WO1).

As a control, two strains were used. One was a normal brewing strain—*Saccharomyces cerevisiae* SafAle™ US-05 (SC)—and the other was a strain designed for low-alcohol beers—*S. cerevisiae* SafBrew™ LA-01 (LA01).

### 2.2. Methods

#### 2.2.1. Wort Preparation and Pitching

The wort (provided by the Korona brewery, Kielce, Poland) used in the research had an extract level of 9.8 °Plato and was hopped using Cascade hops. The wort was sterilized.

The propagation of yeast cultures was carried out according to Cioch-Skoneczny et al. [19].

The pitching rate of yeasts was 10^6^ cells per mL of wort per 1 °Plato. 500 mL of beer wort, and an adequate amount of yeast was added to a fermentation vessel, which was fitted with an airlock and sealed with a parafilm.

#### 2.2.2. Course of the Fermentation

The course of fermentation was determined as in Klimczak et al. [18].

#### 2.2.3. Alcohol and Extract Content

The analysis was performed according to Ciosek et al. [20]. A DMA 4500 M Anton Paar beer analyzer (Anton Paar, Graz, Austria) was used to determine the alcohol content, real and apparent extract content, real and apparent fermentation, and caloric content.

#### 2.2.4. Color

The color of the filtered beer was measured spectrophotometrically, using Beckman DU-650 UV–Vis spectrophotometer (Triad Scientific, Manasquan, NJ, USA) at a wavelength of 430 nm, according to the Analytica EBC 8.5 color of wort spectrophotometric method (IM) [21].

#### 2.2.5. Free Amino Nitrogen Content

Free amino nitrogen (FAN) was measured using ninhydrin-based methods with the use of the absorbance measurement at 570 nm (Beckman DU-650 UV–Vis) according to the EBC method (8.10 free amino nitrogen in wort by spectrophotometry) (IM) [21].

#### 2.2.6. Beer Bitterness

The beer and wort bitterness were determined using an isooctane extraction method with absorbance measurement at 275 nm (Beckman DU-650 UV–Vis) according to the EBC method, 9.8. bitterness of beer (IM) [21].

#### 2.2.7. Turbidity

The beer turbidity was measured using a Cyberscan TN 100 (Merazet, Poznań, Poland) nephelometer, according to EN/ISO 7027 norms [22].

#### 2.2.8. Titratable Acidity and pH

Analysis was performed according to Cioch et al. [23]. The total acidity was calculated as the amount of 1 M NaOH per 100 mL of a beer.

#### 2.2.9. Analysis of Metal Ions

The analysis was performed according to Ciosek et al. [24]. The metal content in the wort was measured using atomic absorption spectrometry with a flame atomization technique (Varian AA240FS—Varian Inc., Palo Alto, CA, USA) and an automatic sample dispensing system (SIPS-20, Agilent, Santa Clara, CA, USA). The gas flow rates were set at 14 dm^2^/min for air and 3.5 dm^2^/min for acetylene. Prior to analysis, samples of wort and beer (3 mL) were placed in sealed pressure vessels with 5 mL of 65% nitric acid added, before undergoing wet mineralization in a Mars Xpress microwave oven (1200 W, 170 °C, 15 min) (CEM Corp., Matthews, NC, USA). After mineralization, the absorbance of the samples was measured at specific wavelengths (422.7 nm for Ca^2+^, 330.2 nm for Na^+^, 213.9 nm for Zn^2+^, and 202.6 nm for Mg^2+^). Metal ions were determined using single-sample aspiration in fast sequential mode. Standard solutions for Ca, Na, Zn, and Mg (50, 50, 5, and 50 mg/L, respectively) were prepared from 1000 mg/L stock solution (Merck, Billerica, MA, USA).

#### 2.2.10. Sensory Analysis

The sensory analysis was conducted by a panel of 8 raters, who had a previous experience in performing beer sensory analysis, were acquainted with the analysis method, and understood the analysis objective. Each of the panelists evaluated the parameters of each of the beers—that is, overall aroma, color, clarity and taste—on a 5-point scale. A score of 1 on the scale equaled the worst, unacceptable quality, whereas 5 equaled the best and most desirable quality.

Additionally, a more detailed aroma analysis was also conducted, which focused on a specific aroma qualities. Each of raters had to specify which of the evaluated aromas was in the beer sample and its intensity. If the aroma was found, its intensity was rated on a 1–5 scale, where 1 equaled a barely noticeable smell and 5 a very strongly perceived smell. If no aroma was detected, its intensity was marked 0. The evaluated aromas were floral, fruity, banana, green apple, herbal, malt, almond, bitter, bready, acetic, clove, solvent, caramel, boiled vegetable, yeasty, milky, acidic, buttery, and mercaptan.

The individual scores for all the quality parameters are presented as an arithmetic mean of all evaluations, presented with the accuracy of 0.1. Additionally, a ‘total’ score was calculated as the sum of the arithmetic means for overall aroma, color, clarity, and taste.

#### 2.2.11. Statistical Analysis

The results are shown as the arithmetic mean of three repetitions, with standard deviation. Furthermore, a repeated-measures ANOVA and Tukey’s (HSD) multiple comparison test were performed at a significance level of α = 0.05. The analyses were performed in Statistica software (StatSoft Poland, Kraków, Poland) version 13.3.

## 3. Results and Discussion

### 3.1. Course of the Fermentation

**Figure 1:**
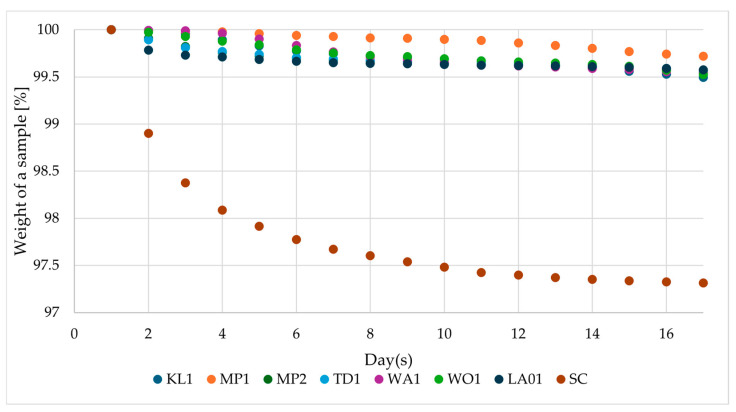
Course of the fermentation of all of the evaluated yeast species. Individual measurements are characterized by a standard deviation below 0.5%.

The fermentation was conducted over 17 days (Figure 1). Based on weight loss, it is clear that *S. cerevisiae* US-05 (SC) carried out the fermentation at the fastest pace. It also fermented the highest amount of sugars from the wort among all the evaluated species. Based on the course of the fermentation, the primary fermentation of SC took place between days 1 and 8. The other yeast species conducted the fermentation more slowly and to a much lower degree (Figure 1).

**Figure 2:**
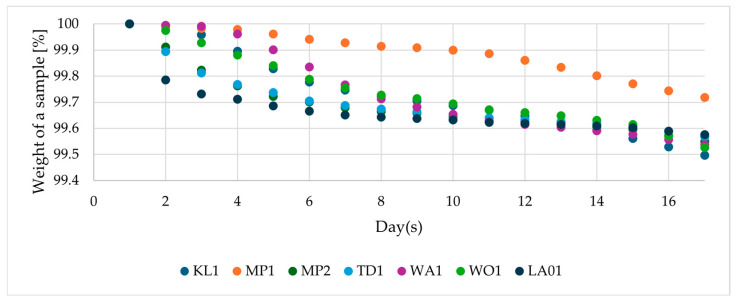
Course of the fermentation of maltose-negative yeast species. Individual measurements are characterized by a standard deviation below 0.5%.

More pronounced differences between the yeast species can be seen in Figure 2. The low-alcohol brewing strain of *S. cerevisiae* (LA01) performed the fermentation at the fastest pace. A similarly fast fermentation course for this strain was reported by González-Siso and Becerra [15]. On days 12–14, the weight losses of beers with different yeasts were at a similar level, except for the MP1 strain. Overall, the MP1 had the slowest fermentation rate. After days 12–14, the fermentation was still continuing slowly for all the evaluated yeast species. However, it was arrested as the fermentation times became unreasonably long. Under industrial conditions, fermentation times for beer can be as short as 3 days for ales and 14 days for lagers. However, these times are highly variable [25,26]. The slow fermentation rate was likely due to lack of maltose utilization [18]. Jackowski et al. [27] reported of *T. delbrueckii* strain with a similar fermentation rate to *S. cerevisiae*, when the wort with reduced maltose content was used. Based on Figure 2, the primary fermentation for the non-*Saccharomyces* yeasts and LA1 lasted from day 1 to days 8–10 of the experiment.

### 3.2. Physicochemical Parameters

The control strain (SC) produced 3.77% (*v*/*v*) ethanol from the 9.8 °Plato wort. All the non-*Saccharomyces* yeasts and LA produced significantly lower quantities of ethanol, in the range of 0.5–1.05% (*v*/*v*). The lowest amounts of alcohol were produced by the MP1 strain, which is consistent with its fermentation course (Figure 2). The other yeast species produced a similar range (0.8–0.9% (*v*/*v*)) of ethanol, with the only exception being the TD1 strain, which produced the highest amount of ethanol among these yeasts (1.05% (*v*/*v*)). Similar low ethanol levels for *T. delbrueckii* strains were found by Michel et al. [28]. However, some *T. delbrueckii* strains are maltose-positive, which can result in much higher alcohol levels [29,30]. Gutiérrez et al. [31] suggest that the *K. lactis* strain evaluated by the authors is promising for the production of fruity beverages, but to the authors’ knowledge, there are no further studies on this yeast species. Many authors report *M. pulcherrima* strains which can at least partially ferment maltose [32,33,34], but the yeast strains evaluated in this study seem to not use this sugar. In a study by Canonico et al. [35], *W. anomalus* produced a beer with 1.5% (*v*/*v*) of alcohol from 12.3 °Plato wort.

Regarding the real extract levels, the results correlate with the alcohol content results. The lowest amount of extract (3.97% (*w*/*w*)) was found in the sample fermented with the SC strain, as it produced the highest amounts of ethanol. The extract content of the low-alcohol beer samples ranged from 8.05 to 8.8% (*w*/*w*). Considering that the wort had an extract of 9.8 °Plato, the low-alcohol strains used only about 1–1.7% of the extract (Table 1).

The ADF (apparent degree of fermentation) of the control strain was 73.4%. This value is relatively low for this strain, and its manufacturer lists higher values (78–82%) [36]. This might have been caused by a higher amount of unfermentable sugars in the wort. The lowest ADF for the rest of the evaluated yeasts was found for the MP1 strain (9.1%), while the highest ADF was observed in the TD1 strain (20.5%) (Table 1).

The wort was characterized by the highest color value of all the samples (30.9 EBC). Throughout the fermentation process, the beer’s color lightened [37]. The lightest color was observed in the SC sample (Table 1). Different yeast species can significantly influence beer color by differing the degree to which the beer lightens [38].

Regarding turbidity, the wort was found to have the highest turbidity of all the samples, at 154 NTU. The samples after fermentation had turbidity levels ranging from 26.2 to 42.5 NTU. There were no statistically significant differences in the turbidity of the beers. The clarity of most of the beers was classified as “brilliant” (<35 NTU), while the clarity of MP1 was classified as almost brilliant (35–69 NTU). The wort was hazy (138–276 NTU) (Table 1) [39,40].

The FAN level of wort was 146 mg/L. According to Hill & Stewart [41], the minimum FAN levels of 10–12 °Plato wort should be in the range of 100–130 mg/L. Lower levels are associated with lagging, incomplete fermentation, and sulfide production [41]. Therefore, the wort had a satisfactory FAN level. The highest utilization of FAN was found in the sample fermented with SC yeast. The beers obtained with low-alcohol yeasts had FAN levels in the range of 111–136 mg/L. Notably, high FAN levels were found for beer obtained with the MP1 strain, which might be linked to its low fermentation ability. High levels of free amino nitrogen in beers can contribute to the formation of haze [42], negatively impact microbial stability [41], or promote the formation of stalling compounds [43,44]. Hence, producing a wort with reduced FAN levels might be beneficial if low-alcohol beer is to be produced. Additionally, this approach might allow for the use of other raw materials with lower FAN levels. Lower FAN usage by the non-*Saccharomyces* yeast, compared to *Saccharomyces* strains, was also reported by Bellut et al. [45].

The wort had a pH of 4.99. Typically, the pH of the brewer’s wort is in the range of 5.0–6.0 [46]. It is known that during fermentation, the pH of the wort decreases, which is attributed to the production of organic acids and the consumption of buffering nitrogen compounds [46]. This decrease can be seen in the SC sample, where the pH of the resulting beer was significantly lower (4.66). Typically, most of the beers have a pH within the range of 4.0 to 4.5 [47]. Interestingly, there was almost no pH drop for most of the produced beers. In some samples, the pH even rose (MP2, TD1, LA) above that of wort. This result was unexpected, as other authors have reported a pH drop, even when very low amounts of alcohol are produced [30,45]. A pH drop was also observed in our previous studies regarding these yeast species [18]. A possible explanation for this fact is the low starting pH of wort. The wort used in the previous study had a much higher pH of 6.05 [18]. This high pH value is one of the reasons for the inappropriate flavor of low-alcohol beers, which contributes to their worty flavor, among other factors [7,26]. An appropriate pH level is also an important hurdle for microbial growth in beer [48,49]. Beer typically has a pH in the range of 4.0–4.5 [47]. Hence, acidification of such beers is often required [7]. High sugar content, low alcohol levels, and high pH are factors that can make low-alcohol beers more susceptible to microbial contamination [50].

Regarding the titratable acidity, the wort had an acidity of 1.48 mL 1 M NaOH/100 mL. The SC sample had a very similar acidity, while the acidity of low-alcohol beers was higher. The highest acidity was found for the WO1 sample. Most of the samples were characterized by similar pH levels, despite having different titratable acidity levels. This is particularly evident in the case of wort, which had the lowest acidity. This is because there is no direct relationship between the pH and titratable acidity [51]. The titratable acidity rises due to the production of weak acids by yeasts. Brewing strains of *S. cerevisiae* are known to produce many organic acids, with the main ones being acetate, pyruvate, and succinate [52,53]. *S. cerevisiae* rapidly acidifies the medium in the absence of organic acids. The lack of significant acidification of the SC sample might be caused by the high content of acids in the wort, which is consistent with its pH [54]. There is no similar information, according to the authors knowledge, regarding non-*Saccharomyces* species. Weak organic acids act as a buffer, preventing pH changes [54,55].

The wort was found to have the highest IBU value. During the fermentation, beer bitterness decreases due to factors such as the drop in pH level and the adhesion of α-acids to yeast cell walls [56]. However, the loss of bitterness varied among the samples. The highest loss was found in the SC sample, which can be at least partially attributed to the highest pH drop after the fermentation. The loss of the bitterness for the other yeasts was much lower, and in some cases, it varied depending on the yeast strain. It is possible that different yeast strains have different affinities for hop bitter acids. Different bitterness levels in resulting beers depending on the strain used have also been reported by Postigo et al. [57] and Rodríguez Madrera et al. [58]. This factor must be kept in mind when using different yeast strains to obtain a beer with the desired bitterness.

### 3.3. Metal Ion Content

The levels of the metal ions in the wort were consistent with the literature data regarding their content in beer (Table 2) [26]. The fermentation led to a reduction in levels of some of the metal ions.

The calcium ions are believed to shield structural membrane phospholipids. Calcium also takes a role as a secondary messenger in the modulation of growth and metabolic responses and is required for flocculation [59]. However, the quantities required for the flocculation are minimal [60]. Significant differences in Ca levels were observed among the beers, with the beer brewed with the typical brewing strain containing the lowest quantity of this metal. The loss of metal ions after the fermentation was greater than that reported by Mochaba et al. [61]. Yeast can accumulate Ca ions during growth and fermentation [62]. However, in one of the beers (MP2), the Ca content was much higher than in the wort, possibly due to ion release from yeast cells. During the fermentation, the Ca, Mg and Zn levels can fluctuate [61,62].

Sodium ion content only differed significantly in the SC sample, where it was considerable lower than in the other beers (Table 2). This suggests that yeast cells may accumulate this ion. Sodium ions exert an inhibitory effect on the growth of yeast and fermentation efficiency [61]. Na levels between 75 and 150 mg/L contribute to the roundness of beer flavor and accentuate sweetness [26].

Zinc is an important trace element, as it acts as co-factor in many of the enzyme classes, governs protein synthesis, and phospholipid composition of membranes. Insufficient wort zinc levels can impair yeast growth and fermentation performance. The zinc requirements are strain-specific [26,63]. The wort had an appropriate level of zinc. Zinc is easily lost in sediment during the preparation of wort. As the wort was sterilized, it may have reduced the zinc content and negatively affected the fermentation performance. However, the wort contained an appropriate zinc level of 1.27 mg/L. The zinc levels in wort should be at least 1 mg/L or 0.15–0.5 mg/L (depending on the source) [26,64,65]. The results showed significant variation in zinc consumption by different yeast strains. Interestingly, it was not a brewing strain of *S. cerevisiae* (SC) that used the highest quantities of these ion, but rather MP1 and WA1 strains (Table 2). Zinc requirements may be strain-specific rather than species-specific, as the two strains of *M. pulcherrima* (MP1 and MP2) exhibited the lowest and the highest zinc utilization, respectively. The requirement for this ion also does not seem to be linked to the alcohol content of the resulting beer, as the TD1 strain produced beer with a highest alcohol content (Table 1) but used one of the lowest quantities of this metal. Despite the literature recommending that zinc levels exceed 1 mg/L, the zinc was not depleted in any of the beers.

Magnesium plays a key role in a variety of biological processes. It is an essential enzyme cofactor and a key structural element of most of biological structures. It activates over 300 enzymes [66,67]. Mg also reduces the stress response of yeast against temperature and ethanol levels [68]. The produced beers had lower levels of magnesium than the wort, with the SC sample showing the lowest concentration (Table 2). This may indicate that SC yeast has the highest demand for magnesium among the examined organisms.

These findings suggest that the evaluated yeast species have different affinities for metals such as Ca, Na, Zn and Mg or may release different quantities of these ions into the beer. However, without yeast enumeration, which was beyond the scope of this study, it is not possible to determine these interactions precisely. 

### 3.4. Sensory Characteristics

**Figure 3:**
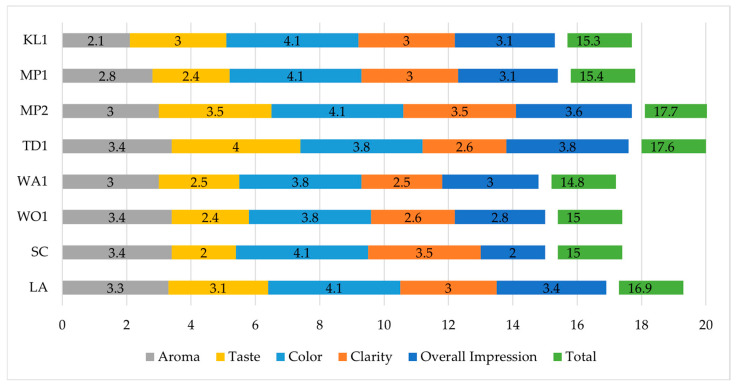
Sensory characteristics of obtained beers.

**Figure 4:**
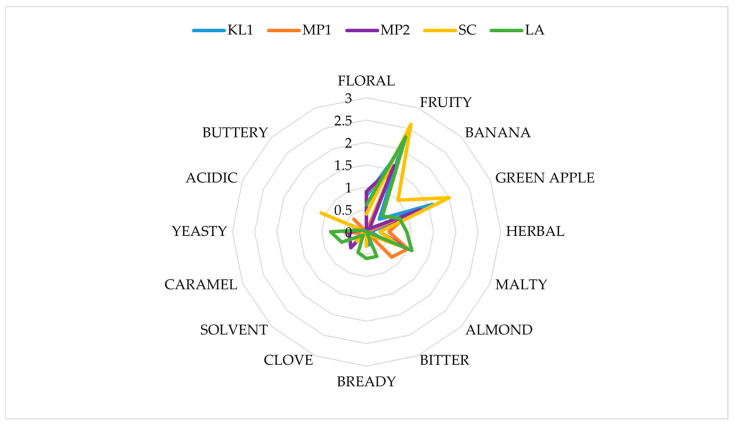
Analysis of beer aroma.

The beers differed in all the discriminants evaluated. In terms of the aroma, the beers produced with non-*Saccharomyces yeasts*, WO1, and TD1 were rated similarly high to the beers produced with *S. cerevisiae*, LA and SC. Other non-*Saccharomyces* beers—WA1, MP2 and MP1—were rated lower. The worst aroma was found in the KL1 sample (Figure 3 and Figure 4). Different yeast species or even strains can produce a different matrix of volatile compounds, which influences the aroma of the beverage [26].

Very significant differences were found in the taste of individual beers. Interestingly, the taste of SC sample was rated the worst among all the beers. The best rated beer, according to the panel, was produced with TD1 yeasts. Beers produced with MP2 and KL1 were also appreciated by the panelists. The commercial strain for low-alcohol beer, LA, was found to produce a beer with an appropriate taste (Figure 3 and Figure 4).

Regarding the color of the beers, the panelists found the color of all the beers to be appropriate (Figure 3 and Figure 4). The differences found using physicochemical analysis were probably not sufficient to influence the panelists’ impression of the beers (Table 1).

Some differences were observed by the panelists regarding the clarity of the produced beers. The samples produced with the SC and MP2 yeasts were rated as having the highest clarity, while WA1, WO1 and TD1 were rated the lowest (Figure 3 and Figure 4). However, it must be noted that the produced beer was not filtered but just transferred from the natural yeast sediment. Typically, in a production environment, the beer is filtered before the next production stages [26].

The overall evaluation of the beers is consistent with previous results. The SC sample was rated the worst among all the samples. This was mostly unexpected, as this strain (SafAle US-05) is widely used in beer production. The panelists noted that the obtained beer was empty in flavor and sour. The commercial strain for low-alcohol beer—LA—received very positive notes. However, the best impression was made by the two non-*Saccharomyces* yeasts, TD1 and MP2. The same trend was observed in the total score for each beer. It must be noted, however, that *T. delbrueckii* ‘lost’ a significant part of its total score due to the low clarity of the produced beer (Figure 3 and Figure 4). This parameter might be optimized in the production environment. *T. delbrueckii* yeasts have long been seen as promising candidates for obtaining uniquely flavored beers and wines [69,70]. The other yeast species which was found interesting—*M. pulcherrima*—has been much less frequently examined with regard to its sensory properties.

These results show that non-*Saccharomyces* yeasts can be used to produce low-alcohol beers with desirable aromas.

**Figure 5:**
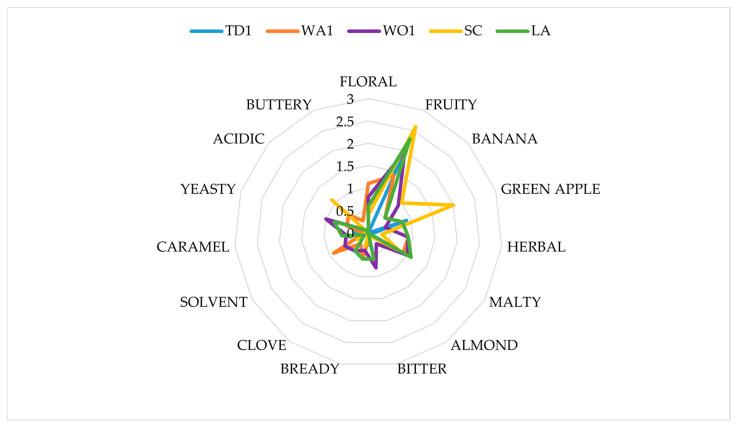
Analysis of beer aroma.

Regarding the individual aroma discriminants, none of the yeasts used produced a beer a with very pronounced aroma (Figure 3 and Figure 4). The panelists did not report the occurrence of acetic, boiled vegetable, milky, and mercaptan aromas in any of the beers, hence they were omitted from Figure 4 and Figure 5.

The SC sample was characterized by the strongest aroma of green apple. This scent is usually seen as a defect. It was probably caused by the high levels of the acetaldehyde, which is described as having a crisp green apple flavor or a grassy one. This compound is an intermediate in the formation of ethanol and acetate, with a sensory threshold of 10–20 mg/L [71]. A probable explanation for such high levels of this compound in US-05 fermented beer is the fact that this beer has the highest ethanol level among all the samples (Table 1). Since acetaldehyde is a precursor for ethanol, its high levels are to be expected. This aroma was also noticeable, albeit to a lower degree, in KL1 and MP2 beers.

Another important discriminant noticed by the panelists was maltiness. This aroma was most noticeable in the LA, WO1, WA1 and MP1 samples. These aromas could be due to elevated levels of aldehydes such as 2-methylbutanal, 3-methylbutanal, and 3-methylthiopropionaldehyde. During the fermentation with normal-alcohol brewing strains of *S. cerevisiae*, these compounds were reduced. The occurrence of these compounds is a problem in some methods of reduced-alcohol beer production [72,73].

A sensory defect, that is, a yeasty smell, was found in LA and WO1 beers, although the aroma intensity was fairly low. An acidic note was found in the SC sample.

All of the beers were characterized by mostly fruity aromas; however, the highest intensity of this aroma was found in both *S. cerevisiae* fermented beers, SC and LA. The second highest intensity was found for KL1, WO1, and TD1. The fruitiness of beers brewed with T. delbrueckii has been documented in the literature [74]. *W. anomalus* is known to impart fruity notes in wine fermentation, and *K. lactis* has been proposed as one of the yeast species that can produce such aromas [11,75].

KL1, MP1, MP2 and WA1 were characterized by a flowery note. Such aromas can be attributed to many groups of compounds such as esters, polyfunctional thiols, lactones and furanones and terpenoids [76]. However, the occurrence of these aromas influences the consumer’s choice of a beer. According to Da Costa Jardim [77], craft beer consumers seem to prefer more aromatic beers with more pronounced fruity aromas.

The low although present banana flavor in SC and WO1 was probably caused by isoamyl and isobutyl acetate [76].

### 3.5. Limitations of the Study

The obtained results show that some of the evaluated yeast strains, such as MP2 or TD1, could be interesting alternative for the production of reduced-alcohol beer. A significant limitation of the study is the lack of optimization of fermentation parameters, such as yeast cell concentration, fermentation time, temperature, or wort parameters, among others. It is possible that if the parameters of a process are optimized, these yeasts might be used in the production of beers which will be appreciated by the average consumer. However, the general aim of the study was to objectively evaluate the yeast strains studied, and further process optimizations are beyond the scope of academic research.

## 4. Conclusions

Non-*Saccharomyces* yeasts are an interesting alternative for the production of reduced-alcohol beer. The evaluated strains differed in their fermentation kinetics, the physicochemical parameters of the produced beers (such as apparent degree of fermentation, IBU, color, and titratable acidity), and the sensory characteristics the of beers. These strains enabled the production of beers with an alcohol content in the range of 0.5–1.05%, depending on the strain. Importantly, the obtained beers had high pH levels; thus, an additional acidification might be necessary. Interestingly, the beers produced using different strains had a significantly different content of evaluated metal ions, which might suggest that they differ in their ability to bind these metals. Among the evaluated strains, *T. delbrueckii* MG971248 and *M. pulcherrima* MG971250 scored the highest in the sensory analysis. These strains were rated even higher than the commercial brewing strains. The results for these strains are promising; however, further research may be necessary. 

## Figures and Tables

**Table 1:** Physicochemical parameters of obtained beers and wort (shown as a mean of three repetitions ± standard deviation).

Yeast Strain	Alcohol [%vol]	Apparent Extract [°P]	ADF [% *w*/*w*]	Real Extract [°P]	Density [g/cm^3^]	Caloric Content [kJ/100 mL]	Color [EBC]	Turbidity [NTU]	FAN [mg/L]	pH	Titratable Acidity [mL 1 M NaOH/100 mL of Beer]	Bitterness [IBU]
KL1	0.80 b (±0.00)	8.05 a (±0.05)	15.1 a (±0.00)	8.35 a (±0.05)	1.03 a (±0.00)	35.1 (±0.00)	26.0 a (±0.27)	32.6 (±5.00)	124 ab (±5.18)	5 ab (±0.06)	1.70 a (±0.07)	20.5 ab (±0.00)
MP1	0.50 a (±0.00)	8.60 d (±0.00)	9.10 b (±0.10)	8.80 d (±0.00)	1.03 d (±0.00	34.4 (±0.00)	25.8 ab (±0.13)	42.5 (±4.25)	136 b (±4.41)	4.95 a (±0.06)	1.69 a (±0.01)	20.5 ab (±0.00)
MP2	0.80 b (±0.00)	8.10 a (±0.00)	15.8 a (±0.10)	8.40 a (±0.00)	1.03 a (±0.00)	34.8 (±0.10)	27.0 a (±0.26)	31.7 (±6.40)	124 ab (±4.95)	5.04 ab (±0.03)	1.73 a (±0.03)	18.7 ac (±0.76)
TD1	1.05 c (±0.05)	7.60 c (±0.20)	20.5 c (±1.90)	8.05 b (±0.15)	1.03 c (±0.00)	34.6 (±0.05)	26.3 a (±1.12)	28.5 (±0.30)	119 ab (±6.62)	5.06 b (±0.03)	1.71 a (±0.09)	20.5 ab (±1.00)
WA1	0.90 b (±0.10)	8.07 a (±0.15)	17.3 a (±1.64)	8.37 a (±0.15)	1.03 a (±0.00)	35.1 (±0.10)	27.1 a (±0.15)	26.5 (±1.95)	129 ab (±9.73)	5.02 ab (±0.02)	1.74 a (±0.04)	18.7 ac (±0.29)
WO1	0.80 b (±0.00)	8.15 a (±0.15)	15.5 a (±0.30)	8.40 a (±0.10)	1.03 a (±0.00)	34.8 (±0.35)	25.9 ab (±0.36)	26.2 (±9.98)	127 ab (±9.95)	5.02 ab (±0.02)	2.18 c (±0.02)	21.7 b (±0.29)
SC	3.77 d (±0.06)	2.63 b (±0.06)	73.4 d (±0.56)	3.97 c (±0.12)	1.01 b (±0.00)	34.8 (±0.70)	24.4 b (±0.36)	34.2 (±14.2)	91.9 c (±1.71)	4.66 c (±0.02)	1.50 b (±0.00)	16.8 c (±0.29)
LA	0.80 b (±0.00)	8.03 a (±0.12)	15.9 a (±0.10)	8.33 ab (±0.12)	1.03 a (±0.00)	34.6 (±0.40)	27.0 a (±0.88)	30.2 (±7.60)	111 a (±5.13)	5.24 d (±0.03)	1.64 ab (±0.07)	19.8 ab (±1.76)
Wort	0.00 (±0.00)	9.80 (±0.00)	0.00 (±0.00)	9.80 (±0.00)	1.04 (±0.00)	35.5 (±0.00)	30.9 (±0.00)	154 (±2.08)	146 (±0.02)	4.99 (±0.00)	1.48 (±0.00)	24.5 (±0.00)

The obtained values were statistically analyzed using an analysis of variance (ANOVA). The results of statistical significance correspond to the interaction of all variables. The absence of assigned letters signifies no statistically significant differences within each group of results at *p* < 0.05. a–d—mean values marked with different letters (for a specific parameter) indicate differentiation as per Tukey’s test (*p* < 0.05).

**Table 2:** Contents of selected ions in produced beers and wort.

	Ca [mg/L]	Na [mg/L]	Zn [mg/L]	Mg [mg/L]
KL1	34.8 a (±1.07)	38.5 a (±0.88)	0.88 abc (±0.12)	77.3 a (±0.87)
MP1	32.5 ac (±1.00)	38.3 a (±0.63)	0.63 a (±0.11)	79.1 a (±1.69)
MP2	61.9 e (±1.98)	39.9 a (±1.29)	1.29 d (±0.04)	89.4 c (±0.58)
TD1	40.9 bd (±0.12)	40.6 a (±1.19)	1.19 cd (±0.16)	85.5 bc (±2.64)
WA1	37.5 ab (±3.4)	43.4 a (±0.61)	0.61 a (±0.13)	81.9 ab (±0.29)
WO1	43.2 d (±2.55)	40.8 a (±1.08)	1.08 bcd (±0.14)	79.4 a (±0.46)
SC	28.6 c (±0.42)	30.4 b (±0.74)	0.74 bc (±0.13)	71.3 d (±3.15)
LA	37.3 ab (±1.44)	41.4 a (±0.92)	0.92 abc (±0.11)	84.9 bc (±2.18)
Wort	48.4 (±0.08)	42.3 (±1.0)	1.27 (±0.18)	97.4 (±0.53)

The obtained values were statistically analyzed using analysis of variance (ANOVA). The results of statistical significance correspond to the interaction of all variables. The absence of assigned letters signifies no statistically significant differences within each group of results at *p* < 0.05. a–e—mean values marked with different letters (for a specific parameter) indicate differentiation as per Tukey’s test (*p* < 0.05).

## Data Availability

The original contributions presented in the study are included in the article; further inquiries can be directed to the corresponding author.
